# The Dental Cosmos

**Published:** 1859-10

**Authors:** 


*The Dental Cosmos.*—This is a monthly publication, edited by Drs. J. D. White, J. H. McQuillen and Geo. J. Ziegler, each No. to contain 56 pages.

The Cosmos takes the place of the Dental News Letter, which has been discontinued. The department of original communications is under the editorial supervision of Dr. White; that of dental literature and art is assigned to Dr. McQuillen; and medicine and general science, in their relations to dentistry, to Dr. Ziegler.

The number before us is handsomely gotten up and contains some excellent original articles, and many judicious selections from other journals.

The names of the editors are a guarantee that the Cosmos will be conducted with ability, and we wish the enterprising publishers, Messrs. Jones & White, abundant success in their new enterprise.

